# Prevalence of Anti-Citrullinated Protein Antibodies (ACPA) in Patients with Diffuse Large B-Cell Lymphoma (DLBCL): A Case-Control Study

**DOI:** 10.1371/journal.pone.0088177

**Published:** 2014-02-07

**Authors:** Gunter Assmann, Klara Shihadeh, Viola Poeschel, Niels Murawski, Jutta Conigliarou, Mei Fang Ong, Michael Pfreundschuh

**Affiliations:** 1 Department of Internal Medicine I, José-Carreras-Centre for Immuno- and Gene Therapy, University Medical School of Saarland, Homburg/Saar, Germany; 2 Department of Internal Medicine, General Hospital of Herrenberg, Esslingen-Herrenberg, Germany; 3 Institute of Medical Biometry, Epidemiology and Medical Informatics, Saarland University, Homburg/Saar, Germany; University of Michigan Medical School, United States of America

## Abstract

**Background:**

Antibodies against citrullinated proteins (ACPA) have been recognised as the most specific serum marker for rheumatoid arthritis. However, serum autoantibodies such as anti-nuclear antibodies have also been detected in the sera of different lymphatic malignancies without accompanying rheumatologic disease. Therefore, we conducted a study to evaluate the prevalence of ACPA in diffuse large B-cell non-Hodgkin lymphoma (DLBCL).

**Methods:**

Sera of 395 DLBCL patients and 258 age-matched healthy controls were investigated to evaluate the prevalence of ACPA and RF. ACPA-positive data were stratified into subgroups of RF positivity and established prognostic parameters for DLBCL, including overall survival. In addition, the ACPA serum concentrations levels were compared to an ACPA-positive RA cohort (n = 175). The statistics were performed with χ2 test and Mann- Whitney-U test; Kaplan-Meyer curves (log rank test) were used to analyse the overall survival. P-value <0.05 was statistically significant.

**Results:**

ACPA, but not RF, occurred significantly more frequently in the sera of DLBCL patients than in healthy controls (3.5% versus 0.8%, p = 0.030). However, the ACPA serum concentration levels were significantly lower than in RA patients (median 10.4 versus 124.1 U/ml, p = 0.0001). After subgroup stratification, ACPA positivity in DLBCL was significantly associated with male gender (4.4% versus 0%, p = 0.022; odds ratio 1.046, CI 1.014–1.079) and with RF-IgM seropositivity (1.77% versus 0%, p = 0.043), but not with prognostic parameters for DLBCL.

**Conclusions:**

DLBCL is associated with a significantly higher prevalence of ACPA, with an increased prevalence in male patients, and simultaneous RF-IgM positivity. However, ACPA is not prognostic for DLBCL. The prevalence of RF-IgM, -IgA, or -IgG did not differ from healthy controls.

## Introduction

Patients with rheumatoid arthritis (RA) are at an increased risk of lymphoproliferative diseases such as myeloma and high-grade B-cell non-Hodgkin lymphoma (B-NHL) compared with the healthy population [1], [2], [3]. The severity and duration as well as anti-rheumatic treatment of RA might further increase this risk [2], [3], [4]. The diminished apoptotic activity as well as the chronic B cell activation mediated through the tumour necrosis factor (TNF) superfamily, such as BAFF (B cell activating factor belonging to the TNF family), is supposed to be the pathogenetic background for this phenomenon [5], [6]. On the other hand, there are no existing data to prove that lymphoma patients are at a higher risk of developing RA. However, serum biomarkers which are established in the diagnostic procedure and prognostic evaluation in RA such as rheumatoid factor (RF) or antinuclear antibodies (ANA) could also be observed in the sera of patients suffering from different forms of lymphatic malignancy.

The rheumatoid factor IgM (of the immunoglobulin M class) is 60–80% positive in RA, but is also positive in up to 50% of non-RA patients suffering from B-lymphocytic leukaemia [7] and additional B-NHL subgroups, such as diffuse-large-B-cell lymphoma (DLBCL), as shown in relatively small cohorts of patients [8]. The antinuclear antibody (ANA) titre also occurs in up to one third of sera derived from RA patients, predicting a severe course of disease and extra-articular manifestations [9]. Several studies have shown that elevated ANA titres are also found in patients with lymphoma [10], [11], [12]. Anti-citrullinated protein antibodies (ACPA) have been reported to be the best diagnostic serum marker with a 99% specificity for RA [13], [14]. Furthermore, numerous longitudinal studies have confirmed that ACPA positivity is associated with a more severe course of RA, particularly concerning the occurrence of bone erosions, compared with ACPA-negative RA patients [15], [16], [17]. Moreover, the study of Nielen et al., investigating serial measurements in healthy blood donors, could show that ACPA is positive in less than 2% of healthy individuals, but, remarkably, ACPA-positive healthy individuals have to be considered to have an evident predisposition for developing RA in the future [18], [19], [20]. Klareskog et al. explained this phenomenon with the hypothesis of an extra-articular “immunisation” of B- and T-lymphocytes being reactive to citrullination, which occurs many years prior to RA manifestation. After that, a “second hit” such as trauma or infection could finally trigger the development of RA [21]. Underlining the predictive capacity of ACPA, a large cohort of primary Sjögren syndrome patients was tested, and 9% of the patients were shown to be ACPA-positive, with more than half of them subsequently developing RA in the following 5 years [22]. Whereas citrullination is basically an ubiquitous process in human cells due to inflammation, previously published experimental models hypothesised that citrullination can also play a role in the regulation of apoptosis through the p53-PAD14 network [14], [23], [24].

The development of ACPA seems to show a link between inflammation and citrullination on one side and autoimmunity and B-cell activation on the other side [18]. Therefore, we conducted a retrospective study analysing the sera of a large cohort of DLBCL patients from the NHLB1/2 study, to evaluate the prevalence of ACPA together with RF-IgM, -IgG, and -IgA in comparison with healthy controls.

## Materials and Methods

### Study Participants

The department of Medicine I at the University Medical School of Saarland is the study centre of the DSNHL German group (Deutsche Studiengruppe Hochmaligne Non-Hodgkin-Lymphome) and conducted the NHLB1 and NHLB2 studies, which recruited 866 and 831 DLBCL patients, respectively [25], [26]. The sera of 395 patients were collected in the laboratory of Medicine I prior to treatment initiation, according to the respective study protocols. Sera from 258 age-matched healthy controls were collected in the Department of Haemostaseology/blood bank and in the Department of Orthopaedics at the University of Saarland; all blood donors were seen by an orthopaedic doctor or rheumatologist to exclude a rheumatologic disorder. Furthermore, 175 ACPA-positive RA patients from the previously published RA study of Assmann et al. were tested for ACPA serum concentrations to compare values with the DLBCL cohort [27]. The study was conducted according to the Helsinki declaration, and was approved by the regional ethics committee (Ethikkommission der Ärztekammer des Saarlandes). All study participants gave their written informed consent. The study population of lymphoma patients was well characterised with respect to all relevant clinical parameters, including previous medical history, stage of lymphoma disease, elevation of serum lactate dehydrogenase (LDH), patients` performance status ECOG (eastern cooperative oncology group) and overall survival (an average [mean] observation period of 6.6 years) using datasheets submitted to the biostatistics institution of Leipzig University. All patients as well as healthy controls suffering from any rheumatic disease or under immunosuppressive treatment or cytostatic medication were excluded from the study. The sera of nine out of 258 healthy controls were not tested for ACPA, but were tested for RF. The sera were collected and stored frozen at −56°C.

### Enzyme Linked Immunosorbent Assay (ELISA) Tests

All serum tests for antibodies against RF-IgM, RF-IgA, RF-IgG, and ACPA-IgG were performed by standard protocols using EUROIMMUN ELISA test sets (EUROIMMUN, Luebeck, Germany). Positive results of ACPA-IgG ranged from 5 to 200 U/ml. All sera were tested twice, and only sera with two positive tests were considered to be positive. RF-IgM results were additionally stratified into subgroups of negative (<50 IU/ml), positive (50–100 IU/ml), and highly positive results (>100 IU/ml).

### Statistics

Differences in age between DLBCL patients and healthy controls were tested by Mann-Whitney test. Differences in gender as well as differences in the frequencies of ACPA and three types of RF between DLBCL and healthy controls were evaluated using χ^2^ test. The ACPA concentration levels were compared by Mann-Whitney-U test. Differences with a p-value of <0.05 were considered significant. All results were corrected by Fisher’s test if required. The overall survival of DLBCL patients stratified into ACPA-positive and ACPA-negative subgroups was tested by Kaplan-Meyer test (log rank test).

## Results

Table 1 shows a summary of the participants’ clinical characteristics. Significantly more male DLBCL patients than male healthy controls compared with female participants required a gender-separated evaluation of the investigated seromarkers. In addition, the sera of 65 DLBCL patients were not determined due to gender.

**Table 1:** Age and gender distribution of DLBCL patients and healthy controls.

	Number	Median age (years)	Gender (%)	
			Male	Female
**DLBCL** *	395	61	55.2**	44.8
**HC**	258	57	46.5	53.5

*p = 0.0192 (difference in age of HC versus DLBCL).

**p = 0.038 (difference in gender distribution of HC versus DLBCL).

DLBCL = diffuse large B-cell non-Hodgkin lymphoma patients.

HC = healthy controls.

DLBCL patients showed a significantly higher prevalence of ACPA compared to healthy controls (3.5% versus 0.8%, p = 0.030; odds ratio [OR] 1.028, confidence interval [CI] 1.005–1.051), whereas all subtypes of RF such as IgM, IgA, and IgG, were equally prevalent in DLBCL patients and healthy controls: RF-IgM 22.8% versus 21.1%; RF-IgA 15.1% versus 12.6%; and RF-IgG 2.8% versus 1.8%. Table 2 shows the serostatus for RF and ACPA in DLBCL patients and healthy controls after stratification by gender. It is evident that the significant difference between DLBCL patients and healthy controls only occurred only in males, but not in females: 4.4% versus 0% (p = 0.022; OR 1.046, CI 1.017–1.079).

**Table 2:** Serostatus for RF and ACPA autoantibodies in DLBCL patients and healthy controls stratified by gender.

		DLBCL			HC				
	M/F	Total number	pos. test	(%)	Total number	pos. test	(%)	p-value	
**RF-IgM pos.**									
(≥50 U/ml)	M	182	40	22	120	26	21.7	0.949	n.s. 1
	F	148	37	25	138	31	22.5	0.615	n.s. 1
**RF-IgM low-pos.**									
(50–100 U/ml)	M	182	9	4.9	120	4	3.3		
**RF-IgM highly-pos.**									
(>100–200 U/ml)	M	182	31	17.0	120	22	18.3	0.776	n.s. 1
**RF-IgM low-pos.**									
(50–100 U/ml)	F	148	14	9.5	138	12	8.7		
**RF-IgM highly-pos.**									
(>100–200 U/ml)	F	148	23	15.5	138	19	13.8	0.879	n.s. 1
**RF-IgA pos.**									
(≥20 U/ml)	M	180	31	17.2	109	12	11.9	0.150	n.s. 1
	F	148	21	14.2	114	16	14.0	0.972	n.s. 1
**RF-IgG pos.**									
(≥20 U/ml)	M	180	7	3.9	109	1	0.9	0.129	n.s. 1
	F	148	3	2.0	114	3	2.6	0.529	n.s. 1
**ACPA-IgG pos.**									
(>5–200 U/ml)	M	182	8	4.4	116	0	0.0	0.022	s. 2*
	F	148	6	3.4	133	2	1.5	0.203	n.s. 1

DLBCL = diffuse large B-cell non-Hodgkin lymphoma patients.

HC = healthy controls.

M = male; F = female.

RF = rheumatoid factor; IgM = immunoglobulin M; ACPA = anti-citrullinated cyclic peptide; pos. = positive;

n.s^1^. = not significant; s. = significant;

*s.^2^ = confidence interval 1.017–1.079; odds ratio = 1.046.

Fourteen out of 395 DLBCL patients versus only two out of 249 healthy controls were ACPA-positive. Table 3 outlines the clinical characteristics of ACPA-positive DLBCL patients (n = 14) compared with ACPA-negative DLBCL patients (n = 381). There was no significant difference in any of the clinical characteristics.

**Table 3:** Clinical characteristics of ACPA positive and negative DLBCL patients.

	ACPA (+)	ACPA (−)	p-value
**Age at diagnosis** (1)	59.4	59.1	0.657
**stage III/IV** (1)	35.5%	40.4%	0.724
**gender (female)** (1)	28.6%	45.1%	0.220
**ECOG >1** (1)	7.1%	11.8%	0.593
**ESR elevated** (1)	21.4%	38.3%	0.200
**LDH elevated** (1)	14.3%	24.2%	0.384
**overall survival** (2) **_(6 years)_**	66.79%	60.26%	0.837

(1) Mann-Whitney U Test.

(2) Log rank/Mantel-Cox tests. ACPA = anti-citrullinated cyclic peptide. ECOG = index of life quality according to the European Cooperative Oncology Group. ESR = erythrocyte sedimentation rate. LDH = lactate dehydrogenase.

**Figure 1:**
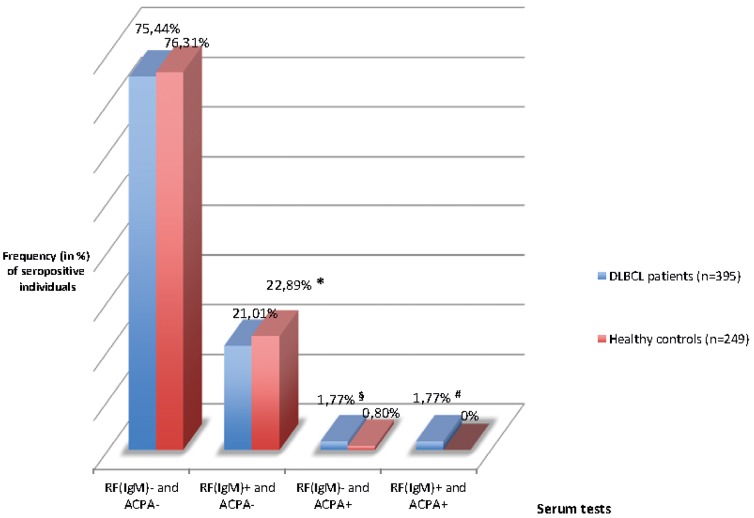
ACPA-positive and -negative DLBCL patients and healthy controls stratified by RF (IgM)-negative and -positive serostatus. DLBCL = diffuse large B-cell non-Hodgkin lymphoma patients. RF−/+ = rheumatoid factor IgM-negative/positive; ACPA−/+ = anti-citrullinated cyclic peptide positive/negative. *no significant difference, p = 0.802. §No significant difference, p = 0.493. ^#^Significantly higher frequency of ACPA in RF (IgM)+DLBCL than in RF(IgM)+HC (p = 0.043).

**Figure 2:**
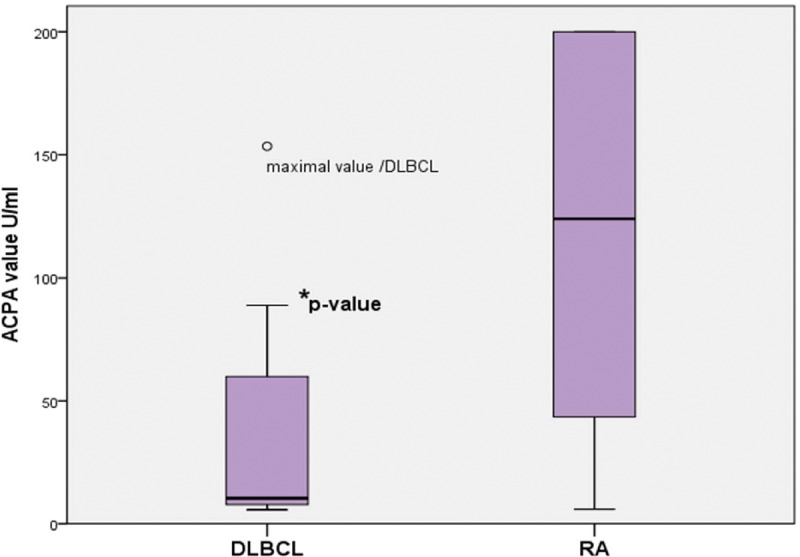
Boxplots presenting results of ACPA serum concentration levels in DLBCL (n = 14) and RA (n = 175). Boxplots with median (10.4 versus 124.1) and first/third quartile, respectively. ACPA−/+ = anti-citrullinated cyclic peptide positive/negative. DLBCL = diffuse large B-cell non-Hodgkin lymphoma patients. *p-value = 0.0001.

Figure 1 shows the subgroup analysis of the study population (DLBCL patients and healthy controls) stratified into RF IgM-positive versus -negative participants. Statistical evaluation of the RF IgM-positive participants (DLBCL patients and healthy controls) showed a significantly higher frequency of ACPA-positive DLBCL patients than healthy controls (1.77% versus 0%, p = 0.043). Among the RF IgM-negative participants, the distribution of ACPA positivity was not significantly different (1.77% versus 0.80%, p = 0.493). Figure 2 shows boxplots presenting the significantly different ACPA concentration levels of DLBCL patients and the RA cohort: median 10.4 (range from 5.7 to 153.4) versus 124.1 (5.9–200); p = 0.0001.

Based on the results shown in table 2 and figure 1, the higher frequency of ACPA positivity in DLBCL patients is associated with male gender and RF-IgM seropositivity. To evaluate the impact of ACPA on overall survival in DLBCL patients, a Kaplan-Meier curve was generated for ACPA+and ACPA - DLBCL patients. There was no significant difference in 6-year overall survival: ACPA +66.79 versus 60.26%, p = 0.837 (log rank, Mantel-Cox, Table 3).

## Discussion

To the best of our knowledge, our study has proved for the first time the higher prevalence of ACPA in DLBCL patients compared to healthy controls. Our results indicate that DLBCL seems to be a lymphatic malignancy with a small subgroup of ACPA-seropositive patients. Next to our study, there has only been one lymphoma study published, in 2010, which has investigated the prevalence of ACPA in B-cell chronic lymphocytic leukaemia, but this showed no ACPA serum positivity [7]. Cambridge et al. reported positive ACPA serum in 10.4% of the 144 patients with coronary heart disease compared to 3.8% of the 288 healthy controls [28]. The study did not investigate a rheumatological or lymphoma patient population, but the authors outlined that the measured ACPA values were relatively lower than those seen in patients with established RA; these results correspond to our data concerning significantly lower ACPA serum concentration levels in DLBCL than in RA. However, Cambridge et al reported a significantly higher prevalence of ACPA in healthy controls compared to most previous studies dealing with the ACPA prevalence e.g. Nielen et al. or Rantapaa-Dahlqvist et al. [19], [20]. The data presented here also showed the commonly low prevalence of ACPA in less than 2% of healthy controls. However, the significance of ACPA in DLBCL remains unclear to date. In particular, ACPA-positive lymphoma patients did not differ in all relevant subgroups, such as ECOG, LDH, stage, and the presence or absence of extranodal lymphoma manifestations, representing the prognostic index. Furthermore, the analysis of overall survival of the DLBCL cohort did not demonstrate a significant difference between ACPA-positive and -negative patients.

Surprisingly, subgroup analysis of ACPA serostatus, stratifying the study population according to the presence of RF-IgM, revealed that ACPA positivity occurred significantly more frequently in RF IgM-positive DLBCL patients than in RF-IgM negative patients. Stratified in this way, the frequency of ACPA positivity in the RF-IgM-negative DLBCL cohort did not differ significantly from RF-IgM-negative healthy controls. These results raise the question whether the development of ACPA in DLBCL could be simply due to a non-specific autoantibody production (of more than one serum autoantibody), as a consequence of malignancy-induced uncontrolled B-cell proliferation in lymphoma. In particular, this hypothesis of unspecific autoantibody proliferation could be supported by the fact that the sera of ACPA-positive DLBCL patients were tested and showed significantly lower ACPA concentrations compared to RA patients.

Performing a further subgroup analysis, we have shown that ACPA-positivity has a significant gender bias, with more male DLBCL patients being positive. To the best of our knowledge, there have been no studies published explicitly investigating the gender distribution of ACPA in RA populations, but large epidemiologic studies, as previously published, have led to the conclusion that no relevant gender-related differences in ACPA positivity exist [29], [30]. In general, several autoimmune diseases such as RA or lupus erythematodes occur significantly more frequently in females than in males [31]. Therefore, our data of lymphoma, showing a preference of male gender in the frequency of RA-related serum markers such as ACPA, would not correspond to previous data of female preference in RA. The general predisposition of female individuals for developing autoimmune diseases was previously discussed in an animal model, demonstrating the higher rate of increased toll-like receptor 7-driven accumulation of CD11+ B lymphocytes in female than in male mice [32]. However, from this point of view, the observation of the accumulation of those subtypes of B lymphocytes in this experimental model appears unsuited to explain, in general, the autoreactivity against citrullinated proteins in DLBCL and, in particular, the preference of male gender, so far.

Regarding citrullination of proteins and development of ACPA, Ireland et al. previously reported an experimental *in vitro* cell culture model including lymphoma cells, where the antigen presentation of citrullinated peptides was not observed in normal B lymphocytes or B lymphoma cells; however, the serum starvation during *in vitro* experiments using B-cell lymphoma cell lines induced the presentation of citrullinated peptides by B lymphoma cells [33], indicating a possible involvement of citrullinated proteins in the autoreactive activity of B lymphoma cells.

Altogether, no convincing link could been found to explain the occurrence of ACPA in DLBCL, so we assumed that the development of ACPA in DLBCL to be preferentially a non-specific immunological phenomenon.

In this situation, one more aspect of ACPA in DLBCL would remain to be elucidated: whether ACPA has the same predictive significance for the development of future RA in the analogy of healthy individuals.

However, despite the relatively large number of DLBCL patients (n = 395) and healthy controls (n = 258), the cohort of only 16 ACPA-positive participants is small. In addition, the follow-up of about six years, as presented here, has to be considered too short for a representative result.

Moreover, the study analysis could been hampered by administered cytostatic treatment including high dosage prednisolone and cyclophosphamide as a possible rheumatic disease-modifying drug, although high dosage cytostatic short-term treatment has been investigated to have no long-term effects on RA in [34],[35].

In conclusion, our study demonstrated a higher prevalence of ACPA in DLBCL patients compared with healthy controls, but our results do not suggest that the targeted clinical application of ACPA evaluation in DLBCL would be useful. With regard to RF-IgM, here we present, to the best of our knowledge, the largest study including gender stratification of DLBCL patients compared with healthy controls, showing no evidence for a higher prevalence of RF-IgM positivity in DLBCL, in contrast to previously published studies. The serum values of RF-IgM which were documented here for healthy controls were higher than in a previously published cohort, but the median age of 57 years in our cohort was higher than in comparable cohorts; furthermore, an inter-assay coefficient of variation for RF-IgM between 4.4% and 16.8% could be previously documented [8], [20].
